# Socioeconomic disadvantage and impact on visual outcomes in patients with viral retinitis and retinal detachment

**DOI:** 10.1186/s12348-022-00303-4

**Published:** 2022-08-02

**Authors:** Ashley Zhou, Sally S. Ong, Ishrat Ahmed, J. Fernando Arevalo, Cindy X. Cai, James T. Handa

**Affiliations:** Wilmer Eye Institute, Johns Hopkins School of Medicine, Baltimore, MD USA

**Keywords:** Social determinants of health, Area deprivation index, Viral retinitis, Retinal detachment

## Abstract

While socioeconomic disparities impact clinical care and patient outcomes, **t**heir impact on the anatomic and visual outcomes of retinal detachment in patients with viral retinitis is unstudied. This case series included 18 eyes in 18 patients from a single academic institution between January 1, 2008 and December 31, 2018. Patient characteristics including age, sex, race, ethnicity, insurance, immunosuppression, viral retinitis, retinal detachment, retinal detachment repair, visual and anatomic outcomes, missed appointments, and Area Deprivation Index [ADI] were collected. The low-ADI group, indicating less socioeconomic disadvantage, was comprised of twelve patients with national ADIs less than 38, and the high-ADI group of six patients with national ADIs greater than 38. High-ADI patients tended to be younger (average age 38.0 versus 51.3; *P* = 0.06), of female sex (*P* = 0.03), and had more missed appointments (median 11.0 vs 0; *P* = 0.002). A similar number of patients in both the high-ADI and low-ADI groups underwent pars plana vitrectomy alone or pars plana vitrectomy with scleral buckle. Visual acuity was similar in the high-ADI group than in the low-ADI group at baseline, but worse at the final follow-up visit (*P* = 0.004). Post-operative and final visit ocular hypotony were more common in the high-ADI group (*P* = 0.02). In our series, socioeconomic disadvantage negatively affects the visual outcomes in patients with viral retinitis associated-retinal detachments. These factors should be considered by ophthalmologists when treating these patients.

**Supplementary Information:**

The online version contains supplementary material available at 10.1186/s12348-022-00303-4.

## Background

Socioeconomic disparities impact many aspects of ophthalmic care, such as access to and utilization of care [1, 2], incidence of ocular pathology [3], choice of treatment approach [4], and visual outcomes [5]. More specifically, socioeconomic disadvantage has been associated with later presentation of common ocular diseases such as cataract, glaucoma, diabetic retinopathy, and age-related macular degeneration [6–10]. The delayed presentation of these ocular pathologies leads to worse vision at baseline and worse visual and anatomic outcomes in the long-run. On the other hand, previous studies have demonstrated that patients with rare diseases have increased compliance and active involvement in their care relative to patients with common diseases [11].

Viral retinitis is a rare but potentially devastating infectious retinal disease that can lead to vision-threatening complications such as retinal detachment. The main pathogens include varicella zoster virus (VZV), herpes simplex virus (HSV), cytomegalovirus (CMV), and Epstein-Barr virus (EBV) [12, 13]. Patients affected by viral retinitis are often immunocompromised, comprising up to 50% of cases [14–16]. Visual outcomes from viral retinitis are generally poor, irrespective of viral etiology [16, 17]. Prompt intervention and careful management may optimize to the extent possible, the visual outcomes from viral retinitis.

Retinal detachment is the leading cause of vision loss in viral retinitis, reported to occur anywhere from 30% to 85% of cases [18–20]. Risk factors include bilateral viral retinitis, the amount of retinal involvement at the time of detachment, and active retinitis near the vitreous base [21–23]. Treatment of retinal detachment typically requires prompt surgical intervention, and is often complicated by atrophic retinal and vitreous changes [24]. Repair options include pars plana vitrectomy, lensectomy, air-fluid exchange, endolaser, scleral buckle, and long-acting gas or silicone oil tamponade [25–32].

Viral retinitis-associated retinal detachment is an uncommon but potentially modifiable visual outcome that is time-sensitive. While the compliance and engagement of patients with viral retinitis-associated retinal detachment based on other uncommon diseases would be predicted to be high, this information is unknown. In fact, the potential adverse impact of socioeconomic factors on uncommon ocular disease such as viral retinitis-associated retinal detachment, its presentation, treatment, and outcomes, is unstudied. Given the potential visual consequences for patients who may already have vulnerable overall health, we studied and describe herein, the extent that socioeconomic disadvantage affects anatomic and visual outcomes for 18 patients with viral retinitis-associated retinal detachments.

## Methods

This case series received approval from the Institutional Review Board at the Johns Hopkins University School of Medicine and adheres to the Declaration of Helsinki and the Health Insurance Portability and Accountability Act. A retrospective chart review was conducted, including records and imaging when available between January 1, 2008 and December 31, 2018 at the Wilmer Eye Institute. The electronic medical record was queried for patients with rhegmatogenous RD using the following codes: ICD-9 codes 361.00, 361.03, 361.02, 361.91 and ICD-10 codes H33.00-, H33.03-, H33.02-, H33.01-; CPT codes 67101, 67105, 67107, 67108, 67110, 67113, 67115, 67120, 67121. This query identified 4974 charts. Inclusion criteria included an age 18 years or older, diagnosis of viral retinitis clinically or by confirmed PCR assay for an etiologic virus in aqueous or vitreous fluid, repair of viral retinitis-related retinal detachment (RD) at the Wilmer Eye Institute, and at least six months of follow-up. Exclusion criteria included age less than 18 years, non-viral retinitis, RD repair at another institution, follow-up of less than six months, and lack of a U.S. zip code due to living outside the U.S. The 4974 charts with diagnoses of RD were manually examined to find patients who fit the inclusion/exclusion criteria, ultimately yielding 18 patients.

Patient characteristics collected included age at diagnosis of viral retinitis, sex, race, ethnicity, insurance, immunosuppression, and missed appointments. Ocular characteristics collected included lens status, intraocular pressure, and the presence of cataract, optic nerve involvement, phthisis, macular scarring or involvement, glaucoma, anterior uveitis, vitritis, vasculitis, papillitis, macular edema, extensive gliosis of optic nerve, macular hole, relative afferent pupillary defect, and keratic precipitate. Viral retinitis characteristics collected included bilaterality, causative virus, location (zonal classification), extension (area), and antiviral medications. Retinal detachment characteristics collected included time to retinal detachment, foveal and macular involvement, extent of detachment, location of detachment (superior, temporal, inferior, nasal), retinal tears, lattice degeneration, presence of proliferative vitreoretinopathy, and any re-detachments. Retinal detachment repair characteristics collected included type of primary surgery (pars plana vitrectomy [PPV], scleral buckle [SB], pars plana vitrectomy with scleral buckle [PPV/SB]), adjuncts (endolaser, cryotherapy, both), use of perfluoro-n-octane, membrane peeling, retinectomy, and tamponade (sulfur hexafluoride [SF_6_], perfluoropropane [C_3_F_8_], silicone oil). Visual outcomes collected included visual acuity (VA) at various time points, primary or secondary reattachment, and complications such as hypotony/increased intraocular pressure, choroidal detachment, residual subretinal fluid, cystoid macular edema, macular pucker, optic atrophy, diplopia, strabismus, and cataract formation.

The socioeconomic status of the patients’ geographic locations on their health outcomes was also evaluated using the Area Deprivation Index (ADI) [33, 34]. The ADI is composed of 17 measures of education, employment, housing-quality, and poverty measures drawn from the 2019 American Community Survey data. Neighborhoods on the nine-digit zip code level are then ranked by ADI score on the national level. A higher ADI indicates a higher level of deprivation and thus a lower socioeconomic status. One patient who resided internationally was excluded from the ADI analysis because the ADI is calculated using US zip-codes. Of the remaining 18 patients, 38 was the average national ADI, and two distinct groups were identified using an ADI threshold of > 38 for the high-ADI and < 38 for the low-ADI group. Six patients were the high-ADI group, or low socioeconomic status, and twelve patients were in the low-ADI, or high socioeconomic status, group.

Two sample t-tests were used to compare the average age at diagnosis of viral retinitis between the low-ADI and high-ADI groups, as well as the average area of viral retinitis. Pearson’s chi-squared was used to compare sex and health insurance status. Wilcoxon rank-sum was used to compare median missed appointments, time to silicone oil removal, baseline and post-operative visual acuity, and time to cataract surgery after primary RD repair. Fisher’s exact was used to compare race, bilateral viral retinitis involvement, bilateral RD, cause of immune dysfunction, causative virus, zonal classification, re-detachment at six months and over follow up, VA changes between baseline and final follow-up, and the number of patients with hypotonic intraocular pressure, cystoid macular edema, macular pucker, optic atrophy, and cataract before and after surgery. Fisher’s exact was also used to compare RD characteristics including whether viral retinitis was active at time of RD, macular involvement, foveal involvement, and proliferative vitreoretinopathy and RD surgery repair characteristics including type of primary surgery, adjuncts, membrane peeling, tamponade, silicone oil removal, and phacoemulsification during silicone oil removal.

## Results

Over a decade, 18 U.S.-based patients who underwent repair of a viral retinitis-associated retinal detachment with at least six months of follow-up were identified. Twelve patients were female and six were male (Table 1). The age at presentation ranged from 29 to 82 years, with an average age of 46.9 years (SD 14.1 years). All but four patients had reported causes of immune dysfunction, including steroids and immunomodulatory medications, as well as systemic illness such as human immunodeficiency virus (HIV), chronic lymphocytic leukemia, myelodysplastic syndrome, non-Hodgkin’s lymphoma, and sarcoidosis. CMV was the most common causative virus, accounting for twelve cases, followed by HSV with four cases, and VZV with two cases. At the time of retinal detachment, the viral retinitis was active in twelve patients. Prior to the retinal detachments, patients took oral acyclovir, valacyclovir, or valganciclovir and were given intravitreal injections of foscarnet and ganciclovir or intravenous ganciclovir.

**Table 1:** Characteristics of all patients with viral retinitis

Case	Age (y)/ Sex	National ADI	ADI group	Insurance type	Missed appointments	Bilateral retinitis	Bilateral RD	Cause of immune dysfunction	Causative virus	Zonal classification	Area of viral retinitis (DAs)	Type of primary surgery	Tamponade
1	59/F	3	Low	No insurance	1	Yes	No	N/A	HSV	2, 3	> 34	PPV	Silicone oil
2	42/F	6	Low	Private	0	No	No	N/A	HSV	3	4	PPV/SB	SF_6_
3	65/M	11	Low	Medicare	0	No	No	Chronic Lymphocytic Leukemia	HSV	2, 3	12	PPV/SB	Silicone oil
4	50/F	15	Low	Private	0	Yes	No	Transplantation, Myelodysplastic syndrome	CMV	3	N/A	PPV	SF_6_
5	29/M	22	Low	Public	0	No	No	HIV	CMV	1, 2, 3	22	PPV	Silicone oil
6	39/M	24	Low	Public	2	Yes	Yes	HIV	CMV	2, 3	18	PPV/SB	Silicone oil
7	59/M	26	Low	Private	3	Yes	No	HIV	CMV	3	6	PPV	SF_6_
8	69/F	27	Low	Medicare	3	No	No	Non-Hodgkin’s Lymphoma	CMV	3	6	PPV/SB	C_3_F_8_
9	82/M	28	Low	Private	0	No	No	Steroids, Sarcoidosis	CMV	3	4	PPV	SF_6_
10	37/F	33	Low	Private	0	No	No	HIV	CMV	2, 3	22	SB	N/A
11	52/F	34	Low	Private	0	Yes	Yes	Steroids	HSV	3	> 30	PPV/SB	Silicone oil
12	50/M	34	Low	Private	1	Yes	Yes	N/A	VZV	3	34	PPV/SB	SF_6_
13	44/F	43	High	Medicare	1	Yes	Yes	HIV	CMV	2	12	PPV	Silicone oil
14	36/F	50	High	Medicare	14	Yes	Yes	HIV	CMV	3	8	PPV/SB	SF_6_
15	40/F	58	High	Public	12	Yes	No	HIV	CMV	1, 2, 3	> 30	PPV	Silicone oil
16	44/F	77	High	Public	13	Yes	No	HIV	CMV	3	> 34	PPV/SB	Silicone oil
17	32/F	91	High	Public	10	No	No	N/A	CMV	2	4	PPV	Silicone oil
18	32/F	99	High	Medicare	10	Yes	Yes	HIV	VZV	3	N/A	PPV/SB	Silicone oil

*ADI* Area Deprivation Index, *HIV* human immunodeficiency virus, *SLE* systemic lupus erythematosus, *CMV* cytomegalovirus, *HSV* herpes simplex virus, *VZV* varicella zoster virus, *DAs* disc areas, *PPV* pars plana vitrectomy, *SB* scleral buckle, *PPV/SB* pars plana vitrectomy with scleral buckle, *SF*_*6*_ sulfur hexafluoride, *C*_*3*_*F*_*8*_ perfluoropropane

The high-ADI group was about a decade younger than the low-ADI group, with average ages of 38.0 (SD 5.5 years) and 51.3 (SD 15.2 years), respectively (*P* = 0.06) (Table 2). All high-ADI patients were female, while the low-ADI group had six male and six female patients (*P* = 0.03). All patients in the high-ADI group had either Medicare or public insurance, while seven low-ADI patients had private insurance (*P* = 0.07). Every patient in the high-ADI group had missed appointments, with a median of 11.0 appointments. Meanwhile, the majority (seven patients) of the low-ADI group had no missed appointments, with a median of 0 missed appointments (*P* = 0.002). Five of the high-ADI group and four of the low-ADI group with missed appointments cited transportation difficulties (*P* = 0.13).

**Table 2:** Baseline demographics

	High ADI	Low ADI	*P* value
Number of patients	6	12	
Age at diagnosis of viral retinitis, mean (SD)	38.0 (5.5)	51.3 (15.2)	0.06
Sex			
Male	0 (0%)	6 (50%)	0.03
Female	6 (100%)	6 (50%)	
Race			
White	1 (17%)	5 (42%)	0.27
Black	5 (83%)	5 (42%)	
Not reported	0 (0%)	2 (17%)	
Insurance type			
Private	0 (0%)	7 (58%)	0.07
Public	3 (50%)	2 (17%)	
Medicare	3 (50%)	2 (17%)	
No insurance	0 (0%)	1 (8%)	
Lost to follow-up	1 (17%)	4 (33%)	1.00
Missed appointments, median (IQR)	11.0 (7.8, 13.3)	0.0 (0.0, 1.8)	0.009
Missed appointments due to transportation issues	5 (83%)	4 (33%)	0.13
Bilateral viral retinitis	5 (83%)	6 (50%)	0.32
Bilateral viral retinitis-associated RD	3 (50%)	3 (25%)	0.34
Cause of immune dysfunction			
Medications	0 (0%)	1 (8%)	0.96
Malignancy Chronic Lymphocytic Leukemia	0 (0%)	1 (8%)	
Non-Hodgkin’s Lymphoma	0 (0%)	1 (8%)	
HIV	5 (83%)	4 (33%)	
Systemic Disorder Myelodysplastic syndrome	0 (0%)	1 (8%)	
Sarcoidosis	0 (0%)	1 (8%)	
None	1 (17%)	3 (25%)	
Patients with HIV	5 (83%)	4 (33%)	0.13
Causative virus			
HSV	0 (0%)	4 (33%)	0.31
VZV	1 (17%)	1 (8%)	
CMV	5 (83%)	7 (58%)	
Area of viral retinitis (DAs), mean (SD)	17.6 (11.8)	17.5 (13.5)	0.99

*ADI* Area Deprivation Index, *RD* retinal detachment, *HIV* human immunodeficiency virus, *CMV* cytomegalovirus, *HSV* herpes simplex virus, *VZV* varicella zoster virus, *DAs* disc areas

All but one of the high-ADI group and a third of the low-ADI group had HIV as the cause of their immune dysfunction (*P* = 0.96) (Table 2). Among the patients with HIV, the high- and low-ADI patients had CD4 counts of 138 (± 200) and 46 (± 56) at presentation, respectively (*P* = 0.40) (Supplementary Table). CMV was the main causative virus for both the high-ADI and low-ADI groups, accounting for all but one of the cases in the high-ADI group and slightly more than half (seven patients) of the cases in the low-ADI group (*P* = 0.31). Bilateral viral retinitis was present in all but one patient in the high-ADI group and half (six patients) of the low-ADI group (*P* = 0.32) (Table 2).

At the time of retinal detachment, viral retinitis was active in all but one of the high-ADI patients and in a little more than half (seven patients) in the low-ADI patients (*P* = 0.60) (Table 3). Pars plana vitrectomy (PPV) and pars plana vitrectomy with scleral buckle (PPV/SB) were used equally in the high-ADI and low-ADI groups (*P* = 1.00) (Table 3). Silicone oil was used for tamponade in all but one of the high-ADI patients, with sulfur hexafluoride (SF_6_) gas used in the remaining patient. In the low-ADI patients, silicone oil and SF_6_ gas were used for five patients each and perfluoropropane (C_3_F_8_) gas for one patient (*P* = 0.51). Silicone oil was not removed in four of the five high-ADI patients. In the low-ADI group, silicone oil was removed in 4 of 5 patients at a median of 6.5 months after the primary surgery.

**Table 3:** Retinal detachment and surgical repair characteristics

	High ADI (*n* = 6)	Low ADI (*n* = 12)	*P* value
Viral retinitis active at time of RD	5 (83%)	7 (58%)	0.60
Macular involvement	4 (67%)	4 (33%)	0.32
Foveal involvement	2 (33%)	3 (25%)	1.00
Proliferative vitreoretinopathy	3 (50%)	3 (25%)	0.34
Type of primary surgery			
PPV	3 (50%)	6 (50%)	1.00
PPV/SB	3 (50%)	5 (42%)	
SB	0 (0%)	1 (8%)	
Other adjuncts			
Endolaser	6 (100%)	11 (92%)	1.00
Cryotherapy	0 (0%)	1 (8%)	
Membrane Peeling	3 (50%)	5 (42%)	0.62
Tamponade			
SF_6_	1 (17%)	5 (42%)	0.51
C_3_F_8_	0 (0%)	1 (8%)	
Silicone oil	5 (83%)	5 (42%)	
None	0 (0%)	1 (8%)	
Silicone oil removal	1 (20%)	4 (80%)	0.21
Time to silicone oil removal, median (IQR)	3.0	6.5 (5.0, 8.0)	0.13
Phacoemulsification + IOL during silicone oil removal	1 (17%)	2 (17%)	0.31

*ADI* Area Deprivation Index, *RD* Retinal detachment, *PPV* pars plana vitrectomy, *PPV/SB* pars plana vitrectomy with scleral buckle, *SB* scleral buckle, *SF*_*6*_ sulfur hexafluoride, *C*_*3*_*F*_*8*_ perfluoropropane, *IOL* intraocular lens

The primary retinal reattachment rate was 83% (five out of six patients) in the high-ADI group and 75% (nine out of twelve patients) in the low-ADI group (Table 4). The baseline preoperative vision was similar in the high-ADI group than in the low-ADI group, with median logMAR equivalents of 1.2 (approximate Snellen equivalent 20/300) and 0.7 (20/100) respectively (*P* = 0.12) (Table 4). In contrast, the postoperative visual recovery was poorer in the high-ADI group relative to the low-ADI group at all the post-operative visits: 6 months (*P* = 0.13), 1 year (*P* = 0.05), and at the final visit, with median final visit logMAR equivalents of 2.6 (20/8000) and 0.7 (20/100), respectively (*P* = 0.004). Baseline vision was not available for one patient in the low-ADI group. Of the six patients in the high-ADI group, one gained 15 + letters, while one lost 10–14 letters, and four lost 15 + letters at final follow-up. Of the twelve patients in the low-ADI group, four gained 15 + letters, one gained 10–14 letters, three gained/lost less than 9 letters, one lost 10–14 letters, and three lost 15 + letters. Two of the patients in the high-ADI group were left with no light perception.

**Table 4:** Anatomic and visual outcomes

	High ADI (*n* = 6)	Low ADI (*n* = 12)	*P* value
Re-detachment at six months	0 (0%)	1 (8%)	0.06
Re-detachment over follow up	1 (17%)	3 (25%)	0.27
VA LogMAR equivalent, median (IQR)			
Baseline	1.2 (1.0, 2.7)	0.7 (0.1, 2.3)	0.12
6-month post-op	1.2 (0.9, 2.1)	0.7 (0.5, 1.1)	0.13
1-year post-op	1.1 (0.8, 2.6)	0.5 (0.2, 0.8)	0.05
Final follow-up	2.6 (2.3, 3.2)	0.7 (0.3, 1.0)	0.004
VA changes between baseline and final follow-up			
Gained 15 + letters	1 (17%)	4 (33%)	0.41
Gained 10–14 letters	0 (0%)	1 (8%)	
Gained/lost less than 9 letters	0 (0%)	3 (25%)	
Lost 10–14 letters	1 (17%)	1 (8%)	
Lost 15 + letters	4 (67%)	3 (25%)	

*ADI* Area Deprivation Index, *VA* visual acuity

Postoperative complications were observed in both ADI groups (Table 5). While cystoid macular edema, macular pucker, and optic atrophy developed with similar frequency at the final visit, hypotony, defined as IOP < 5 mmHg, was statistically distinct between the high- and low-ADI groups. Four of six high-ADI patients experienced new-onset postoperative hypotony compared to one of twelve patients in the low-ADI group (*P* = 0.02). Two of the four high-ADI patients had proliferative vitreoretinopathy that may have contributed to their hypotony. Importantly, hypotony remained in three of four high-ADI patients at the final visit and was associated with either light perception or no light perception vision. In contrast, one low-ADI patient had hypotony that resolved by the final visit. Five of the six patients in the high-ADI group had cataracts prior to the primary RD repair (*P* = 0.02), and the sixth patient developed cataract after the procedure (Table 5). These cataracts were visually significant.

**Table 5:** Postop complications

	High ADI (*n* = 6)	Low ADI (*n* = 12)	*P* value
Hypotonic intraocular pressure (< 5 mm Hg)	4 (67%)	1 (8%)	0.02
Cystoid macular edema	1 (17%)	5 (42%)	1.00
Macular pucker	2 (33%)	3 (25%)	1.00
Optic atrophy	1 (17%)	1 (8%)	1.00
Cataract present before surgery	5 (83%)	2 (17%)	0.02
Cataract formation after surgery	0 (0%)	5 (42%)	0.11
Timing cataract surgery after primary RD repair, median (IQR)	3.0	7.5 (6.0, 10.0)	0.16

*ADI* Area Deprivation Index, *RD* retinal detachment

## Discussion

Viral retinitis is uncommon. Retinal detachment is a rare, but devastating complication of viral retinitis. At a busy academic Retina center, only 18 patients developed viral retinitis-associated retinal detachment over a decade. When it does occur, viral retinitis can be devastating, especially with retinal detachment, which can contribute to irreversible vision loss. In our series, viral retinitis severity and retinal detachment characteristics were similar between the high- and low-ADI groups. Specifically, the preoperative visual acuity, viral retinitis profile, RD characteristics, surgical approach, postoperative complications, and single surgery reattachment rate were similar between the high- and low-ADI groups. Likewise, the immunocompromised state, bilateral viral retinitis involvement, area of viral retinitis, and CMV as the viral pathogen especially with retinal detachment, which are associated with poor outcome, were similar between groups [35]. However, patients with high ADIs and thus high socioeconomic disadvantage had more missed appointments and worse visual outcome. Our results contrast a previous study that demonstrated higher re-detachment rates in patients with socioeconomic disadvantage and rhegmatogenous retinal detachment not exclusive to viral retinitis [36]. This difference in outcome may be due to the viral etiology of our study as opposed to the variety of retinal detachment etiologies in the study by Moussa et al. (2021).

Ultimately, patients with higher ADIs were vulnerable due to sociodemographic factors. All of the high-ADI patients were female, and not more likely to be non-White or underinsured. Importantly, the all female high-ADI patients had significantly more missed appointments. These findings agree in part with previous studies that identified low-income, racial minority, female sex, underinsured, and chronically ill patients with more missed appointments, more medical comorbidities, limited health access, and increased morbidity and mortality [37, 38]. Unlike patients with other uncommon disorders [11], the high ADI-status negatively impacted the compliance of patients with the rare condition of viral retinitis-associated retinal detachment.

The majority of noncompliant patients in our study cited difficulties with transportation to their appointments. The cost of missed appointments, specifically due to transportation issues, increases both patient morbidity and medical costs, particularly for vulnerable patients [39]. National health care studies show that patients who lack access to nonemergency medical transportation are disproportionately female and can be clustered in certain areas, like the patients in this case series [40]. We believe that these missed appointments due to gender inequity and the patient’s neighborhood characteristics had a detrimental impact on the final visual outcome due, for example, to suboptimal monitoring of anti-inflammatory and anti-viral medicine.

Despite advances in medical care and policy interventions, socioeconomic disparity is likely to persist, making it crucial that clinicians keep these factors in mind when providing optimal ophthalmological care for patients with a severe disease like viral retinitis. In the future, to improve compliance, consideration should be given to provide transportation to patients with clearly identified need and to offer scheduling flexibility for patients with life stressors, another reason cited for missed appointments. The provision of nonemergency medical transport through healthcare rideshare applications has been demonstrated to effectively reduce no-show rates in patients [41]. Given that missed appointments are associated with more costly medical care and acute care utilization, providing transportation to patients could be a cost-effective strategy to improve continuity of care [42, 43].

In addition, patient education that includes making patients aware of missed appointments, the impact of missed appointments on patients’ health and the clinic, negotiating a commitment to improved adherence, and modified double-booking such as booking both morning and afternoon slots in order to optimize patient flow, have been shown to improve patient compliance, and should be designed into patient management, especially for high-ADI patients [44]. Furthermore, HIV was the main causative immunosuppression in high-ADI patients. Given the chronic and complex course of viral retinitis and the underlying immunosuppressive conditions, these patients would benefit from close and regular follow-up. Interventions based on education and assistance have reduced gender inequities in all-cause blindness, clinic attendance, and treatment coverage, and should be considered when designing treatment for patients with viral retinitis-associated retinal detachments.

Limitations of this study include its retrospective nature, which potentially introduced bias and affected the availability of data in the electronic medical record over the decade. For example, visual acuity was impacted by ocular comorbidities such as cataract and some visual acuity measurements, imaging modalities were not available for some patients at certain time points, and because the first presentations of some of the patients were their retinal detachment repair, information characterizing the treatment and course of the viral retinitis prior was not available. Furthermore, statistical analysis and the power of the study were limited by its small sample size due to the rarity of viral retinitis-associated retinal detachments. The Area Deprivation Index (ADI) was used as a proxy for socioeconomic status, but this measure is limited to zip-code level analysis. Though subregional and individual variation are certainly possible, ADI has been validated and has the advantage of including factors such as income, education, employment, and housing quality [34]. Previous health outcomes studies, including those evaluating retinal detachments, have utilized similar regionally derived measures of deprivation [10, 45–47].

## Conclusions

It is clear that socioeconomic disadvantage and gender disparities negatively affect the clinical course and the anatomic and visual outcomes in patients with viral retinitis-associated retinal detachments. Further studies are required not just in the context of viral retinitis, but also to explore the multitude of ways in which socioeconomic factors can impact ophthalmological care and the ways in which healthcare systems can mitigate these impacts. In patients with viral retinitis and retinal detachments, retina specialists need to pay close attention to socioeconomic factors and gender because they can influence patient compliance and treatment outcomes.

## Supplementary Information

**Additional file 1: Supplementary table.** Subgroup analysis for patients with HIV.

## Data Availability

The datasets used and/or analysed during the current study are available from the corresponding author on reasonable request.

## Abbreviations

ADI: Area Deprivation Index
VZV: Varicella zoster virus
HSV: Herpes simplex virus
CMV: Cytomegalovirus
EBV: Ebstein-Barr virus
ARN: Acute retinal necrosis
PORN: Progressive outer retinal necrosis
AIDS: Acquired immunideficiency syndrome
cART: Combination antiretroviral therapy
RD: Retinal detachment
PPV: Pars plana vitrectomy
SB: Scleral buckle
PPV/SB: Pars plana vitrectomy with scleral buckle
VA: Visual acuity
SF_6_: Sulfur hexafluoride
C_3_F_8_: Perfluoropropane
HIV: Human immunodeficiency virus
SLE: Systemic lupus erythematosus
DAs: Disc areas
IOL: Intraocular lens
