# Supplementary material for: Socioeconomic disadvantage and impact on visual outcomes in patients with viral retinitis and retinal detachment
Source: J Ophthalmic Inflamm Infect. 2022 Aug 2;12:26. doi: 10.1186/s12348-022-00303-4 (PMC9346012; doi:10.1186/s12348-022-00303-4)
Supplement: Supplementary file 1 — Additional file 1: Supplementary table. Subgroup analysis for patients with HIV. [file 12348_2022_303_MOESM1_ESM.docx]

Supplementary table: Subgroup analysis for patients with HIV

|  | High ADI | Low ADI | P value |
| --- | --- | --- | --- |
| Number of patients | 5 | 4 |  |
| CD4 count at diagnosis | 138 (200) | 46 (56) | 0.40 |
| Age at diagnosis of viral retinitis, mean (SD) | 39.2 (5.2) | 42.2 (12.5) | 0.64 |
| Sex |  |  |  |
| Male | 0 (0%) | 3 (75%) | 0.05* |
| Female | 5 (100%) | 1 (25%) |  |
| Race |  |  |  |
| White | 1 (20%) | 1 (25%) | 0.68 |
| Black | 4 (80%) | 2 (50%) |  |
| Not reported | 0 (0%) | 1 (25%) |  |
| Insurance type |  |  |  |
| Private | 0 (0%) | 2 (50%) | 0.13 |
| Public | 2 (40%) | 2 (50%) |  |
| Medicare | 3 (60%) | 0 (0%) |  |
| No insurance | 0 (0%) | 0 (0%) |  |
| Lost to follow-up | 0 (0%) | 1 (25%) | 1.00 |
| Missed appointments, median (IQR) | 5 (1.5, 9) | 0.0 (0.0, 2.3) | 0.09 |
| Missed appointments due to transportation issues | 4 (80%) | 1 (25%) | 0.21 |
| Bilateral viral retinitis | 5 (100%) | 2 (50%) | 0.17 |
| Bilateral viral retinitis-associated RD | 3 (60%) | 1 (25%) | 0.52 |
| Causative virus |  |  |  |
| HSV | 0 (0%) | 0 (0%) | 1.00 |
| VZV | 1 (20%) | 0 (0%) |  |
| CMV | 4 (80%) | 4 (100%) |  |
| Area of viral retinitis (DAs), mean (SD) | 21.0 (12.9) | 17.0 (7.6) | 0.60 |
| Viral retinitis active at time of RD | 4 (80%) | 3 (75%) | 1.00 |
| Macular involvement | 3 (60%) | 1 (25%) | 0.52 |
| Foveal involvement | 1 (20%) | 0 (0%) | 1.00 |
| Proliferative vitreoretinopathy | 3 (60%) | 0 (0%) | 0.17 |
| Type of primary surgery |  |  |  |
| PPV | 2 (40%) | 2 (50%) | 0.71 |
| PPV/SB | 3 (60%) | 1 (25%) |  |
| SB | 0 (0%) | 1 (25%) |  |
| Other adjuncts |  |  |  |
| Endolaser | 5 (100%) | 3 (75%) | 0.44 |
| Cryotherapy | 0 (0%) | 1 (25%) |  |
| Membrane Peeling | 3 (60%) | 2 (50%) | 1.00 |
| Tamponade |  |  |  |
| SF6 | 1 (20%) | 1 (25%) | 0.68 |
| C3F8 | 0 (0%) | 0 (0%) |  |
| Silicone oil | 4 (80%) | 2 (50%) |  |
| None | 0 (0%) | 1 (25%) |  |
| Silicone oil removal | 1 (20%) | 2 (5%) | 0.52 |
| Time to silicone oil removal, mean (SD) | 3.0 | 8.5 (2.1) | 0.07 |
| Phacoemulsification + IOL during silicone oil removal | 1 (20%) | 1 (25%) | 1.00 |
| Redetachment at six months | 0 (0%) | 1 (25%) | 0.44 |
| Redetachment over follow up | 1 (20%) | 2 (50%) | 0.52 |
| VA LogMAR equivalent, median (IQR) |  |  |  |
| Baseline | 1.3 (0.9, 2.8) | 0.4 (0.0, 0.8) | 0.03* |
| 6-month post-op | 1.3 (0.8, 2.5) | 0.6 (0.5, 0.8) | 0.11 |
| 1-year post-op | 1.1 (0.8, 2.6) | 0.5 (0.3, 0.7) | 0.10 |
| Final follow-up | 2.9 (1.6, 3.2) | 0.6 (0.0, 1.7) | 0.07 |
| VA changes between baseline and final follow-up |  |  |  |
| Gained 15+ letters | 1 (20%) | 1 (25%) | 1.00 |
| Gained 10-14 letters | 0 (0%) | 0 (0%) |  |
| Gained/lost less than 9 letters | 0 (0%) | 1 (25%) |  |
| Lost 10-14 letters | 1 (20%) | 0 (0%) |  |
| Lost 15+ letters | 3 (60%) | 2 (50%) |  |
| Hypotonic intraocular pressure (<5 mm Hg) | 4 (67%) | 0 (0%) | 0.33 |
| Cystoid macular edema | 1 (20%) | 1 (25%) | 1.00 |
| Macular pucker | 1 (20%) | 0 (0%) | 0.52 |
| Optic atrophy | 1 (20%) | 0 (0%) | 0.52 |
| Cataract present before surgery | 5 (100%) | 0 (0%) | 0.01* |
| Cataract formation after surgery | 0 (0%) | 2 (50%) | 0.17 |
| Timing cataract surgery after primary RD repair, average (SD) | N/A | 9.5 (3.5) |  |

ADI: Area Deprivation Index; HIV: human immunodeficiency virus; SLE: systemic lupus erythematosus; CMV: cytomegalovirus; HSV: herpes simplex virus; VZV: varicella zoster virus; DAs: disc areas; PPV: pars plana vitrectomy; SB: scleral buckle; PPV/SB: pars plana vitrectomy with scleral buckle; SF_6_: sulfur hexafluoride; C_3_F_8_: perfluoropropane; RD: retinal detachment; IOL: intraocular lens; VA: visual acuity
